# An Analysis of Reported Dangerous Incidents, Exposures, and Near Misses amongst Army Soldiers

**DOI:** 10.3390/ijerph15081605

**Published:** 2018-07-28

**Authors:** Ben Schram, Robin Orr, Timothy Rigby, Rodney Pope

**Affiliations:** 1 Tactical Research Unit, Bond University, Robina, QLD 4226, Australia; bschram@bond.edu.au (B.S.); rpope@csu.edu.au (R.P.); 2 Faculty of Health Science and Medicine, Bond University, Gold Coast, QLD 4226, Australia; trigby@student.bond.edu.au; 3 School of Community Health, Charles Sturt University, Albury-Wodonga, NSW 2640, Australia

**Keywords:** full-time, part-time, military, tactical, army, reserve, risk, army, incidents

## Abstract

Occupational health and safety incidents occurring in the military context are of great concern to personnel and commanders. Incidents such as “dangerous incidents”, “exposures”, and “near misses” (as distinct from injuries, illnesses, and fatalities) indicate serious health and safety risks faced by military personnel, even if they do not cause immediate harm. These risks may give rise to harm in the future, if not adequately addressed, and in some cases the incidents may cause latent harm. The purpose of this study was to ascertain the rates and patterns of incidents of these types reported by full time (ARA) and part time (ARES) Australian Army personnel. A retrospective cohort study was performed using self-reported incident data from the Workplace Health, Safety, Compensation and Reporting (WHSCAR) database over a two-year period. Data were analysed descriptively. Of 3791 such incidents, 3636 (96 percent) occurred in ARA and 155 (4 percent) in ARES personnel, somewhat consistent with the proportions of total army person-years served in each (ARA 93 percent; ARES 7 percent). In ARA, 84 percent of these incident types were exposures, 14 percent near misses, and 2 percent dangerous incidents. In ARES, 55 percent of incidents were exposures, 38 percent near misses, and 7 percent dangerous incidents. Soldiers at the rank of ‘private’ experienced the highest rates of these incident types, in both ARA and ARES. Driving gave rise to more near misses than any other activity, in both populations. Exposures to chemicals and sounds were more common in the ARA than ARES. The ARES reported higher proportions of vehicle near misses and multiple mechanism dangerous incidents than the ARA. The findings of this study can usefully inform development of risk mitigation strategies for dangerous incidents, exposures, and near misses in army personnel.

## 1. Introduction

The occupational requirements of military personnel during training and operations include intense combat training, vigorous manual handling, patrolling and direct combat, and these job requirements can expose personnel to extremely dangerous situations and incidents [1]. While many such situations and incidents result in physical injuries or illnesses [2], some incidents comprise “dangerous incidents”, “exposures”, or “near misses”—outcomes that indicate the health of the soldier and potentially the military team and mission have been placed at serious risk [1,3].

“Dangerous incidents” are defined by the Australian Department of Defence as incidents that have exposed the worker or any other person to a serious risk to their health or safety, emanating from an immediate or imminent exposure to an uncontrolled release of a hazard [4]. Examples can include exposure to explosions, electrical shocks, collapsed structures, or interruption of air supply [4]. “Exposure” is defined as actual or potential exposure to hazardous substances or materials, or a traumatic event, which has not resulted in any immediate effects on any person and was not immediate or imminent and so did not meet the definition of a “dangerous occurrence” [4]. These tend to be exposures that are less serious or better controlled, and might include, for example, airborne or contact exposures to chemicals or heat arising from fires, which might have been substantially controlled or protected against by use of personal protective equipment [3,4,5,6,7]. Importantly, both dangerous incidents and exposures may cause latent harm, which due to their latency have not yet been recognized or reported as occupational injuries or illnesses. “Near misses” are incidents that could have, but did not result in serious injury to, or death of, a worker or other person, and which did not expose any person to an immediate or imminent risk [4]. An example of a near miss might include the collapse of a structure that people often walk under or climb, but which no-one was near at the time the incident occurred [3,4].

Hazardous environmental exposures reported by veterans from operations in Iraq and Afghanistan include dust storms, smoke from burning trash, oil fires, vehicle exhaust, chemicals, and petrochemical fuel exposures [8]. A retrospective analysis showed that almost all (94 percent) of veterans of these two conflicts reported hazardous exposures during their deployment [9]. More broadly across all services in the military, other investigations reported 25 percent had experienced a dangerous incident—such as exposure to biological warfare agents, oil fires, or witnessing a traumatic event—at some stage during their military careers [10]. An additional 53 percent of military personnel reported exposure to hazardous or dangerous occurrences that required use of protective equipment, such as respirators or hearing protection [10]. Other studies have identified that lethal weapons operation, the operation of sophisticated weaponry systems, military motor vehicle incidents, and working under extreme environmental conditions are associated with high numbers of reported hazardous exposures in deployed U.S. personnel [3,11].

The long-term health ramifications of dangerous incidents and exposures are concerning [5,6,12]. Exposure to dangerous and hazardous substances such as biological warfare agents and asbestos in the Gulf War has led to higher than usual incidence rates of hypertension, musculoskeletal problems, traumatic stress disorder, and periodontal disease when affected personnel are compared to non-deployed troops [13]. Exposures to high caliber weapons, especially in built up areas, during active service has led to high rates of moderate to severe hearing loss [14]. Exposure to air borne hazards such as volatile organic compounds and particulate matter produced from burning solid waste pits, oil fires, and dust storms has led to respiratory complaints including respiratory illness, decreased forced vital capacity, and other long-term health problems [5,10,15,16].

Considering this, while documented evidence of hazardous exposures is widely available in the literature [3,5,6,8,9,10,11,12], data on ‘near miss’ incidents have been more difficult to obtain, as most of the literature focusses on dangerous and hazardous exposures that cause immediate or latent health problems [3]. Reporting near misses may serve to reduce instances of exposures and incidents, highlighting the importance of creating a workplace environment which encourages near miss reporting as collaborative as opposed to punitive [17]. Furthermore, while some literature is available discussing differences in injury rates and patterns between reserve (part time) and regular (full time) soldiers [2,18,19,20], there is no known literature comparing rates and patterns of dangerous incidents, exposures, or near miss incidents in these two army populations.

As military operations increase, the likelihood of such incidents occurring increases in parallel [21]. Given that reserve forces now make up around 10 percent of Australian [22] and United Kingdom forces [23], and about half of United States military personnel actually engaged in current conflicts [24,25], this specific sub-population within army forces warrants dedicated consideration. The need for such consideration is predicated on the requirement for these reserve personnel to complete the same duties as full time forces [26] while typically having a primary employment outside the military [18,23] and being exposed to less military training than full time forces [27]. As such, the purpose of this research was to investigate the rates and patterns of dangerous incidents, exposures, and near misses reported by Australian Regular Army (ARA) and Australian Army Reserve (ARES) personnel and to compare these rates and patterns across ARA and ARES populations.

## 2. Materials and Methods

### 2.1. Study Design

A retrospective cohort study was conducted to provide a detailed description of the rates and patterns of “dangerous incidents”, “exposures”, and “near misses” experienced and reported by ARA and ARES soldiers. Incidents of these types that were reported over a two-year period (1 July 2012 to 30 June 2014) by both ARA and ARES personnel were sourced from the Workplace Health, Safety, Compensation and Reporting (WHSCAR) database of the Australian Department of Defence and constituted the basis for this study.

### 2.2. Data Collection and Participants

The WHSCAR database is designed to record all of the workplace health and safety incidents that both ARA and ARES personnel report as having occurred while they were on duty during service in the Australian Army. Once collected, each WHSCAR record is classified by incident type, as representing a “minor personal injury” (MPI), “serious personal injury” (SPI), “fatality”, “dangerous incident”, “exposure”, or “near miss”, in accordance with the Australian Department of Defence, Work Health and Safety Event definitions [4]. In addition, data including the activity description, service type (permanent army or army reserve), incident location (demographic location), rank of affected person, age range description, incident summary, nature of injury (if applicable), body location of injury (if applicable), activity description (training, operations or combat), mechanism of incident, and agency description are also categorised.

For this specific study, which investigated “dangerous incidents”, “exposures”, and “near misses”—rather than injuries, illnesses, or fatalities—records extracted from the WHSCAR database were included in the study if they represented an incident that: (a) affected ARES or ARA personnel; (b) occurred when the soldiers were on duty; (c) occurred between 1 July 2012 and 30 June 2014; and (d) was classified as either a dangerous incident, exposure, or near miss. Records were excluded if the incident: (a) affected (or potentially affected) only a non-human member of the army (for example, a canine member); (b) affected only people other than soldiers; or (c) were classified as an injury, illness, or fatality.

### 2.3. Data Extraction

All injury and incident data were extracted from the WHSCAR database by a third party WHSCAR data operator, specifically trained in the use of this database and independent of the research team. Following extraction, non-identifiable data, were provided to the research team in a Microsoft Excel spreadsheet. The research team then screened the data records, applying the inclusion and exclusion criteria discussed in the preceding section. All included incident records were grouped by type of incident (“dangerous incident”, “exposure”, or “near miss”) and by service type (ARA or ARES). Ages reported in the incident records ranged from 17 to 63 and were grouped to form eight separate age range categories. These age categories were chosen to allow for comparison of the findings from this study with results of previous research [28,29].

The mean population sizes of both ARES and ARA were drawn from published Department of Defence records [30,31]. The total number of days of active service undertaken by ARES personnel, as a cohort, in the study period was provided by administrators of the army’s personnel databases and reflected actual days worked by ARES personnel during that period.

The Australian Defence Human Research Ethics Committee (ADHREC, Protocol LERP 14-024) and the Bond University Human Research Ethics Committee (BUHREC, RO1907) provided ethics approval for this study. Departmental approval to conduct the research was gained as part of the ADHREC ethics approval process. Authorization to publish this research was provided by Joint Health Command.

### 2.4. Data Analysis

The data were imported into the Statistical Package for the Social Sciences (SPSS) software (IBM 2015, SPSS Inc., Chicago, IL, USA), version 21.0, for statistical analysis. Descriptive analyses were first performed to examine, describe and better organize the data. Specific incident mechanisms were summarized separately if those mechanisms were reported in more than one percent of total incidents, whereas any mechanism reported in less than one percent of incident records was pooled into an ‘other’ classification. Annual per-capita rates of the incidents of interest in both ARA and ARES populations were calculated by dividing the total numbers of reported incidents that occurred across the two-year period in each service type by two, to get a mean annual number of incidents, and then dividing this mean number of incidents by the mean number of personnel in the service type across the two-year period. These per capita incidence rates for ARA and ARES populations were then each multiplied by 100 to derive mean annual incident rates per 100 personnel, for both the ARA and ARES populations.

Additionally, the total numbers of incidents of interest that were reported across the two-year study period were each in turn divided by the total number of years of active service provided to the army by personnel from each population (ARES and ARA), across the two-year study period, to derive incidence rates reported in terms of incidents per 100 person-years of active service (i.e., full-time equivalent years). When calculating total years of active service (i.e., total full-time equivalent years of service) for the ARES, 232 days of active service were assumed to equate to one full year of active service (or one full-time equivalent year of service) based on the following calculation:

Total days of active service typically completed in a full-time year of army service = 365 days in a year, minus 104 days on weekends (or “in lieu” non-service days), minus 20 days of annual leave, minus 9 days of public holidays.

A population estimate of the ARES: ARA incidence rate ratio (IRR) for the incidents of interest, indicating the ratio of incidence rates in ARES compared to ARA, was calculated using the following formula [32]:

IRR = (ARES incidence rate)/(ARA incidence rate)(1)

In these IRR calculations, the incidence rates used were those based on total number of fulltime equivalent years of active service (rather than total number of personnel). The 95 percent confidence interval (95% CI) around the population estimate of each IRR was then calculated as [32]:

95% CI = exp (ln[IRR] − 1.96 × SE(ln[IRR])) to exp (ln[IRR] + 1.96 × SE(ln[IRR]))(2)

where

SE(ln[IRR]) = √(1/[incidentrateARES] + 1/[incident rateARA] − 1/nARES − 1/nARA)(3)

## 3. Results

In total, 3791 records of “dangerous incidents”, “exposures”, and “near misses” that had occurred within the two-year study period were eligible for inclusion in the study. Based on this number and taking into account the population sizes listed in Table 1, the overall army incidence rate for the reported incidents of interest was 4.3 reported incidents per 100 personnel each year. Among these 3791 reported incidents of interest, 3636 (96 percent) involved ARA personnel and 155 (4 percent) involved ARES personnel, giving respective ARA and ARES incidence rates of 6.2 and 0.5 reported incidents of these types per 100 personnel each year, when the population sizes listed in Table 1 are considered. However, when expressed as incidents per 100 person-years of service (Table 1), the ARA and ARES incidence rates were, respectively, 6.2 and 3.3 reported incidents of these types per 100 person-years of service. This result indicated that the combined rates of these types of reported incidents were almost twice as high in the ARA as in the ARES when the numbers of days actually served were considered, with a reported incident rate ratio for ARA versus ARES of 1.9 (95% CI 1.6 to 2.2).

The frequencies and incidence rates for reported incidents of each type (dangerous occurrence, near miss, or dangerous incidents) are shown in Table 2. The most common incident type across the army was exposures (82 percent of all incident types), followed by dangerous incidents (15 percent) and near misses (3 percent).

The distributions by rank of incidents of the three types of interest in both the ARES and ARA are indicated in Table 3. The highest numbers of incidents of these types occurred in those at the rank of private in the ARA and ARES. Consistent with the data presented in Table 2, exposures were the most common incident type, followed by dangerous incidents and near misses, across all ranks except recruits, where dangerous incidents were more commonly reported than exposures or near misses.

The activities in which incidents of the three types of interest occurred to ARA and ARES personnel can be seen in Table 4 and Table 5 respectively. Incidents of these types occurring during operational activities were more prevalent in the ARA than in the ARES, representing 58.1 percent of all such incidents in the ARA and most commonly involving exposures. Operational dangerous incidents in the ARA were typically due to detonations of improvised explosive devices in proximity of personnel, while the one near miss was due to an encounter with a snake whilst on operation. Common types of exposure experienced whilst ARA personnel were performing operational duties included exposure to asbestos during Operations RESOLUTE (border protection and security operation) and ASTUTE (stabilisation operations in support of Timor-Leste), exposure to loud noise through small arms fire, and general exposure to environmental hazards in Afghanistan, such as smoke from burning oil. Of concern is the unknown activity type reported for many incidents (Table 4 and Table 5). This classification was used in reports of 18.7 percent of all such incidents in the ARES and 4.9 percent of all such incidents in the ARA, providing little detail on particulars of these incidents.

The activity type accounting for the greatest proportion of total incidents in ARES personnel was driving related activity, mostly resulting in exposures (Table 5). These exposures were most commonly exposure to hazardous chemicals, including gases and fumes, whilst a passenger in a vehicle. Where driving related activity resulted in near misses, these incidents were generally noted to have been minor accidents with no reported injuries. Driving related dangerous incidents were commonly due to vehicle roll overs, impact with animals or other vehicles, or parts failure. Incidents occurring during operational activities were less common in the ARES population, accounting for only 7.1 percent of all incidents of interest in this service type; all of these were reported to have involved an exposure (Table 5).

The mechanisms by which incidents of the three types of interest occurred in ARA and ARES populations can be found in Table 6 and Table 7, respectively. The reported incidents amongst ARA personnel most commonly occurred due to long term contact/exposure to chemicals and single contact with chemicals. These ARA exposures to chemicals or substances were most commonly exposure to asbestos, industrial fumes and gasses, or environmental hazards like dust whilst on deployment.

The mechanism by which incidents of the three types of interest most commonly occurred in ARES personnel was a single contact with chemicals, with all such incidents listed as exposures (Table 7). These single contacts with chemicals mostly involved exposure to hazardous chemicals whilst a passenger in a vehicle, consistent with the driving related incident predominance in Table 5. Other commonly reported mechanisms by which incidents of the three types of interest occurred in ARES personnel included multiple mechanisms (24.5 percent), by which dangerous incidents most commonly occurred, and vehicle accidents (16.8 percent), which were associated with near misses and dangerous incidents (Table 7).

**Figure 1:**
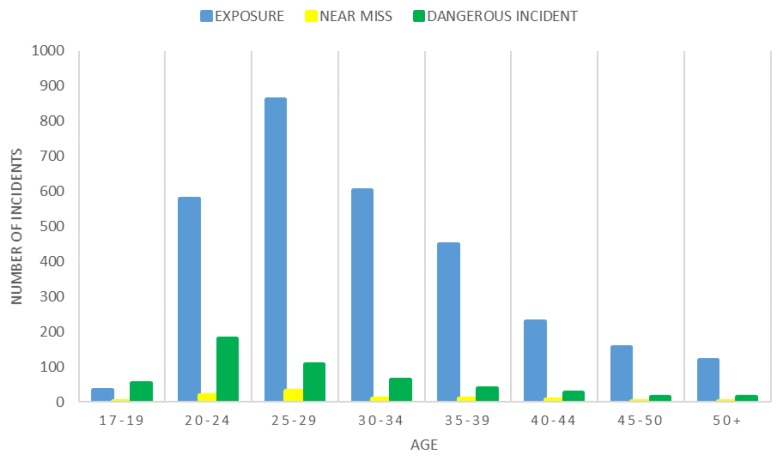
Incidents occurring in different age groups in the ARA.

**Figure 2:**
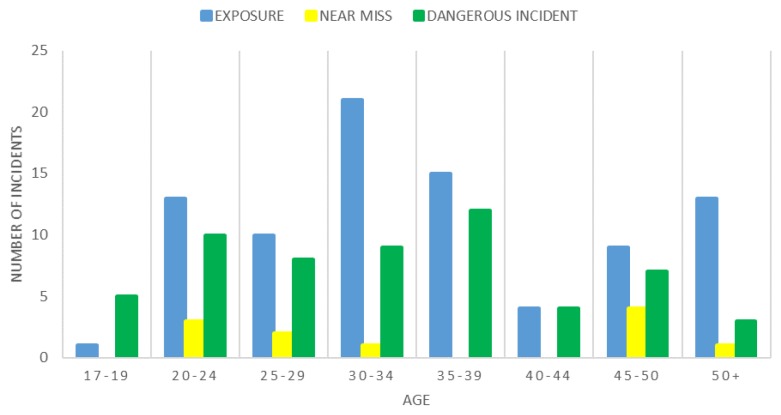
Incidents occurring in different age groups in the ARES.

The distributions of the three types of incidents of interest in ARA and ARES personnel across age categories are displayed in Figure 1 and Figure 2, respectively. Exposures were most prevalent in the 25–29 years age bracket in the ARA, whereas dangerous incidents were more common in the 20–24 years age bracket. Exposures were more common in the 30–34 years age bracket in ARES, while dangerous incidents peaked in the 35–39 years age bracket.

## 4. Discussion

The purpose of this study was to investigate the rates and patterns of dangerous incidents, exposures, and near misses reported by ARA and ARES personnel and to compare these rates and patterns across ARA and ARES populations. Across the army as a whole, 3791 such incidents occurred during the two-year study period, giving an incidence rate of approximately 6 incidents of these types per 100 years of active service. However, there were differences between ARA and ARES populations in rates of these types of incidents. An incident of one of these types took place 6.2 times in every 100 years of active service in the ARA, but at around half that rate (3.3 incidents per 100 years of active service) in the ARES. Breaking these rates down by incident type, the ARA was observed to have a threefold higher rate of exposures than the ARES, and exposures accounted for 84 percent of all such incidents in the ARA, but the ARES had slightly higher rates of dangerous occurrences and near misses than the ARA. With respect to the high rate of exposures in the ARA, closer scrutiny revealed that 77 percent of these were reported as long term or single exposures to chemicals, which in turn most commonly involved exposure to asbestos or environmental hazards like dust whilst on deployment, as will be further discussed below. The higher rates of near misses in ARES may be a true increased risk for those who are only working in this environment part time and therefore more likely to make a mistake. Conversely, they may be more likely to report a near miss, given the culture of their primary occupation, or the fact that they are not entitled to free ongoing health care—unlike their full time colleagues—and are therefore more likely to report incidents [33]. In line with the results from this study, previous reports have found a greater number of injuries reported from reserve personnel when compared to full time personnel [34].

### 4.1. Rank

With respect to rank, 63 percent of all reported exposures, dangerous occurrences and near misses occurred in the ranks of private and corporal. While relative numbers of personnel at these rank levels are likely to have contributed to this finding, the finding is consistent with previous studies that have shown rank to influence the risk faced by army personnel [35,36]. Private and corporal ranks are suggested as being at a higher risk of incidents due to the roles, capabilities, and responsibilities of these ranks [36]. As private-ranked soldiers are newly qualified soldiers who have often only recently completed basic training, they may be at greater risk due to lower levels of experience than their higher ranked colleagues. This lack of experience may make it more difficult for them to recognize and manage incident risks and perhaps give rise to more risk-taking behaviour [23]. The low incident rates observed in the recruit ranks (Table 2) may be due in part to less exposure to incident-prone tasks, such as deployment, as they were still in a training phase of their overall military career and so in more controlled situations and under careful scrutiny and guidance. The large drop in incident rates in ranks above major is likely attributed to the relatively low numbers of higher ranked officers. Unfortunately, no profile data were available to allow for comparisons across ranks based on relative numbers of personnel at each level.

### 4.2. Activity

Exposures whilst on operations featured heavily in the ARA incidents, but much less in the ARES. Operational incidents accounted for two-thirds of all exposures in the ARA, and dangerous incidents were most prevalent in weapons firing activities and near misses were more commonly associated with driving-related activities. Driving related activities were the most common activity type being performed at the times incidents occurred in the ARES, with the majority of near misses and over a quarter of both exposures and dangerous incidents in the ARES occurring during driving-related activities. Previous studies have also noted high risks of military motor vehicle driving related incidents amongst army personnel [11,37,38]. Previous research has reported that up to 57 percent of active duty army personnel took part in risky driving behaviour such as speeding, non-seatbelt use, and alcoholic intake that resulted in a near miss incident [11]. Previous studies of the military suggest that, while typically military personnel are younger and fitter than the general population, they are more likely to take part in risky behaviour such as heavy drinking then their civilian counterparts, exposing them to greater risk and exposure to driving related incidents [39,40]. In the US military, 11 percent of non-battle injuries and 44 percent of non-battle fatalities have been have been attributed to motor vehicle accidents whilst on deployment [41].

Firing weapons was the leading activity in which dangerous incidents occurred in both the ARES and ARA, accounting for 41 percent and 37 percent of all dangerous incidents reported in ARES and ARA, respectively. Weapons handling practices are a major threat to the safety of military personnel, with the US military 2008 injury prevention report finding that 10 percent of non-battle fatalities in Operation Iraqi Freedom and 18 percent of non-battle fatalities in Operation Enduring Freedom were due to the handling of weapons and explosives [41].

Of concern, and of note for future recording of incidents within Defence Forces, was the fact that the unknown activity description was associated with five percent of all incidents of the types of interest in the ARA, the second most common activity category associated with incidents of these types in the ARES and the leading activity category associated with exposures in the ARES. This may be due to insufficient details of the incidents being provided or available when they are reported retrospectively, or insufficient coding in the WHSCAR database to capture adequate details of the incidents.

### 4.3. Mechanisms

Long term and single event contact or exposures to chemicals were the leading mechanism for incidents of the types of interest in the ARA population, accounting for 65 percent of all three incident types and 77 percent of exposures. Similarly, these two mechanisms were features of 46 percent of all incidents of the types of interest in the ARES population and 84 percent of all exposures. Previous studies have also noted high prevalences of exposures to chemicals, with studies reporting more than half of 77,047 U.S military personnel required protective equipment for chemicals, airborne hazards, and hearing hazards during military service [9,10,13,15]. Of more concern, other studies have shown high rates of chemical warfare agent exposure in the Gulf War and Operation Iraqi Freedom [13]. The high rates of exposure to chemicals is alarming considering the documented long term physical and mental health effects of such exposures, including heart disease, cancer, periodontal disease, and musculoskeletal disorders [5,6,9,10,15]. Soldiers who have undergone high levels of combat stress are thought to be to be up to seven times more likely to believe they have been exposed to chemical agents [42]. Together, these findings not only highlight the threat of chemical exposure to defence force personnel, but also reinforces the importance of stress management.

Exposure to sound, or noise, also featured as a mechanism involved in incidents of the types of interest, particularly in the ARA where it accounted for six percent of the incidents of interest. The chronic exposure to sound or noise amongst full time personnel is consistent with previous research, with some studies finding hearing loss in up to 55 percent of soldiers, with another 18 percent suffering severe hearing loss [14]. This exposure to sound is thought to reflect a high frequency of combat situations, use of high caliber weapons, fighting in built up areas, and participation in military training exercises [43,44].

### 4.4. Age

The highest numbers of incidents of the types of interest in the ARA were found in the 25–29 years old age group and were most commonly exposures. Conversely, ARES personnel reported more incidents in the older age bracket of 30–34 years old, again most commonly exposures. This result could be reflective of the age difference between the two as in the Australian Army, full time personnel are typically younger than reservist personnel (median age 29 vs. 41 [45]). These higher incidents in lower age brackets is however in agreement with other studies which have found an increase in risk taking behaviour and decreased mental maturity in younger military populations [28,29]. Older soldiers tend to have lower injury rates, which may also be a result of a higher rank with a longer career, leading to sedentary, staff, or supervisory positions with less field time and subsequently less risk of experiencing incidents. This finding of an increased rate of incidents in younger military members may highlight the need to identify and target incidents that are a result of controllable risk-taking behaviours evident early in a soldier’s career when they may not have the experience required to minimize these risks.

Clearly there are some factors which may increase risk of exposures, dangerous incidents and near misses. The geographic location of both deployment and training may expose soldiers to airborne particulate matter, asbestos, high vehicle emissions and burn pits [10]. Personal characteristics of the individual soldier, including smoking, alcohol use and not wearing seatbelts, has been shown to further augment risk and enhance susceptibility to incidents [11,29]. Optimizing physical characteristics such as body composition and muscle strength has been shown to increase the ability of soldiers to withstand severe environmental conditions and better cope with malfunctioning equipment [29,46].

### 4.5. Limitations

There is a limitation to this study which should be acknowledged. It should be noted that this research is based on retrospective self-reported data and consequently there is potential for recall error (recall bias) or for incidents to go unreported and as such may be under representative of the dangerous incidents, exposures, and near misses experienced by this population. Given that the current system requires the individual to report the incident to a system (and not a person), it may not capture the full picture that a user-friendly hybrid system utilising both point of care and soldier/supervisor reporting approaches might.

## 5. Conclusions

It is evident that substantially more exposures—but not near misses or dangerous incidents—were reported in the study period in the ARA than in the ARES. Common exposures that are inevitable with operational deployment are more prevalent in the ARA, and these include exposures to operational combat, weapons firing, and long-term exposure to sounds and chemicals. In the ARES, exposures, near misses, and dangerous incidents commonly occur during driving-related activities, perhaps due to the lower levels of military experience of ARES personnel.

## Figures and Tables

**Table 1:** Numbers (%) of army personnel, person-years of service and incidents, by service type.

	ARA	ARES	COMBINED
Personnel (2012–2014)	29,401 (66%)	15,034 (34%)	44,435 (100%)
Person-years of service	58,802 (93%)	4701 (7%)	63,503 (100%)
Total incidents	3636 (96%)	155 (4%)	3791 (100%)

**Table 2:** Frequencies of incidents and incidence rates (incidents per 100 soldiers per year (per 100 years of active service)), by service type.

Incident Type	ARA		ARES		Combined	
	*n* (%)	**Incidence Rate**	*n* (%)	Incidence Rate	*n* (%)	Incidence Rate
Exposure	3038 (84%)	5.17 [5.17]	86 (55%)	0.29 [1.83]	3124 (82%)	3.52 [4.92]
Dangerous incident	507 (14%)	0.86 [0.86]	58 (38%)	0.19 [1.23]	565 (15%)	0.64 [0.89]
Near miss	91 (2%)	0.15 [0.15]	11 (7%)	0.04 [0.23]	102 (3%)	0.11 [0.16]
**Total**	**3636 (100%)**	**6.18 [6.18]**	**155 (100%)**	**0.52 [3.29]**	**3791 (100%)**	**4.27 [5.97]**

**Table 3:** Reported incident frequencies by rank.

Rank	ARA	ARES	Combined
Recruit	95 (2.6%)	9 (5.8%)	104 (2.7%)
Private	1350 (37.1%)	47 (30.3%)	1397 (36.9%)
Corporal	967 (26.6%)	39 (25.2%)	1006 (26.5%)
Sergeant	351 (9.7%)	9 (5.8%)	360 (9.5%)
Warrant officer	239 (6.6%)	11 (7.1%)	250 (6.6%)
Officer cadet	41 (1.1%)	21 (13.5%)	62 (1.6%)
Lieutenant	134 (3.7%)	4 (2.6%)	138 (3.7%)
Captain	247 (6.8%)	9 (5.8%)	256 (6.8%)
Major	151 (4.2%)	4 (2.6%)	155 (4.1%)
Lieutenant colonel	46 (1.3%)	0 (0%)	46 (1.2%)
Colonel	7 (0.2%)	0 (0%)	7 (0.2%)
Brigadier	3 (0.1%)	0 (0%)	3 (0.1%)
Major general	1 (0.03%)	0 (0%)	1 (>0.1%)
Unknown	4 (0.1%)	0 (0%)	4 (0.1%)
**Total**	**3636 (100%)**	**153 (100%)**	**3789 (100%)**

**Table 4:** Activities in which exposures, dangerous incidents, and near misses occurred in ARA personnel.

Activity Description	Dangerous Incident	Exposure	Near Miss	% of Total Incidents
Operational	16 (3.2%)	2102 (68.8%)	1 (1.1%)	2119 (58.1%)
Weapons firing	187 (37.3%)	85 (2.8%)	5 (5.5%)	277 (7.6%)
Equipment maintenance	20 (4.0%)	213 (7.0%)	9 (9.9%)	242 (6.6%)
Driving Related	121 (24.1%)	43 (1.4%)	25 (27.5%)	189 (5.2%)
Unknown	25 (5.0%)	149 (4.9%)	4 (4.4%)	178 (4.9%)
Combat training	36 (7.2%)	112 (3.7%)	16 (17.6%)	164 (4.5%)
Manual handling	24 (4.8)	70 (2.3%)	11 (12.0%)	105 (2.9%)
Flying/aircraft movement	4 (0.80%)	64 (2.1%)	0 (0%)	68 (1.9%)
Clerical	0 (0%)	70 (2.3%)	0 (0%)	70 (1.9%)
Parachuting	14 (2.8%)	18 (0.6%)	5 (5.5%)	37 (1.0%)
Patrolling	14 (2.8%)	15 (0.5%)	7 (7.7%)	36 (1.0%)
Chemical handling	0 (0%)	30 (1.0%)	0 (0%)	30 (0.8%)
Other	41 (8.2%)	85 (2.8%)	8 (8.8%)	134 (3.7%)
**Total**	**502 (100%)**	**3056 (100%)**	**91 (100%)**	**3649 (100%)**

**Table 5:** Activities in which exposures, dangerous incidents, and near misses occurred in ARES personnel.

Activity Description	Dangerous Incident	Exposure	Near Miss	Total Incidents
Driving related	17 (29.3%)	24 (27.9%)	7 (63.6%)	48 (31.0%)
Unknown	1 (1.7%)	26 (30.2%)	2 (18.2%)	29 (18.7%)
Weapons firing	24 (41.4%)	2 (2.3%)	0 (0%)	26 (16.8%)
Combat training	7 (12.1%)	6 (6.9%)	1 (9.1%)	14 (9.0%)
Operational	0 (0%)	11 (12.7%)	0 (0%)	11 (7.1%)
Equipment maintenance	0 (0%)	7 (8.1%)	1 (9.1%)	8 (5.2%)
Patrolling	4 (6.9%)	1 (1.2%)	0 (0%)	5 (3.2%)
Other	5 (8.6%)	9 (10.4%)	0 (0%)	14 (9.0%)
**Total**	**58 (100%)**	**86 (100%)**	**11 (100%)**	**155 (100%)**

**Table 6:** Mechanisms by which exposures, dangerous incidents, and near misses occurred in ARA personnel.

Mechanism	Dangerous Incident	Exposure	Near Miss	Total Incidents
Long term contact/exposure to chemicals or substances	0 (0%)	1620 (53.3%)	0 (0%)	1620 (44.6%)
Single contact with chemicals or substances	9 (0%)	707 (23.3%)	3 (3.3%)	719 (19.8%)
Multiple mechanisms	277 (54.6%)	4 (0.1%)	47 (51.6%)	328 (9.0%)
Long term exposure to sounds	0 (0%)	209 (6.9%)	0 (0%)	209 (5.7%)
Contact to biological factors (unknown origin)	0 (0%)	169 (5.6%)	0 (0%)	169 (4.6%)
Vehicle accident	120 (23.7%)	1 (0.03%)	30 (33%)	151 (4.2%)
Contact with biological factors (non-human origin)	0%	84 (2.8%)	0 (0%)	84 (2.3%)
Other environmental factors	0%	105 (3.5%)	0 (0%)	105 (2.9%)
Contact with biological factors (human origin)	0%	28 (0.9%)	0 (0%)	28 (0.8%)
Explosion	33 (6.5%)	3 (0.1%)	0 (0%)	36 (1.0%)
Other	68 (13.4%)	108 (3.6%)	11 (12.1%)	187 (5.1%)
**Total**	**507 (100%)**	**3038 (100%)**	**91 (100%)**	**3636 (100%)**

**Table 7:** Mechanisms by which exposures, dangerous incidents, and near misses occurred in ARES personnel.

Mechanism	Dangerous Incident	Exposure	Near Miss	Total Incidents
Single contact with chemicals or substances	0 (0%)	65 (75.6%)	0 (0%)	65 (41.9%)
Multiple mechanisms	37 (63.8%)	1 (1.2%)	0 (0%)	38 (24.5%)
Vehicle accident	18 (31.0%)	0 (0%)	8 (72.7%)	26 (16.8%)
Long term contact/exposure to chemicals or substances	0 (0%)	7 (8.1%)	0%	7 (4.5%)
Contact with hot objects and electricity	1 (1.7%)	1 (1.2%)	2 (18.2%)	4 (2.6%)
Exposure to non-ionizing radiation	0 (0%)	3 (3.5%)	0%	3 (1.9%)
Exposure to sounds	0 (0%)	3 (3.5%)	0%	3 (1.9%)
Contact with biological factors (human origin)	0 (0%)	2 (2.3%)	0%	2 (1.3%)
Fall	0 (0%)	1 (1.2%)	1 (9.1%)	2 (1.3%)
Other	2 (3.4%)	3 (3.5%)	0 (0%)	5 (3.2%)
**Total**	**58 (100%)**	**86 (100%)**	**11 (100%)**	**155 (100%)**
